# Haploinsufficiency Interactions between RALBP1 and p53 in ERBB2 and PyVT Models of Mouse Mammary Carcinogenesis

**DOI:** 10.3390/cancers13133329

**Published:** 2021-07-02

**Authors:** Sharda P. Singh, Jihyun Lee, Chhanda Bose, Hongzhi Li, Yate-Ching Yuan, Ashly Hindle, Sharad S. Singhal, Jonathan Kopel, Philip T. Palade, Catherine Jones, Rakhshanda L. Rahman, Sanjay Awasthi

**Affiliations:** 1 Division of Hematology & Oncology, Department of Internal Medicine, Texas Tech University Health Sciences Center, Lubbock, TX 79430, USA; jihyun.lee@ttuhsc.edu (J.L.); chhanda.bose@ttuhsc.edu (C.B.); ashly.hindle@ttuhsc.edu (A.H.); jonathan.kopel@ttuhsc.edu (J.K.); catherine.jones@ttuhsc.edu (C.J.); rakhshanda.rahman@ttuhsc.edu (R.L.R.); 2 Department of Surgery, School of Medicine, Texas Tech University Health Sciences Center, Lubbock, TX 79430, USA; 3 Bioinformatics Core Facility, City of Hope National Medical Center, Duarte, CA 91010, USA; holi@coh.org (H.L.); yyuan@coh.org (Y.-C.Y.); ssinghal@coh.org (S.S.S.); 4 Department of Medical Oncology, City of Hope National Medical Center, Duarte, CA 91010, USA; 5 Department of Pharmacology and Toxicology, University of Arkansas for Medical Sciences, Little Rock, AR 72205, USA; ppalade@uams.edu; 6 UMC Health System, UMC Cancer Center, Lubbock, TX 79415, USA

**Keywords:** RalBP1, breast cancer, metastasis, Erbb2, PyVT

## Abstract

**Simple Summary:**

Rlip knockout has been reported to prevent cancer in highly cancer-susceptible mice lacking p53, and Rlip knockdown kills many types of cancer cells. In humans, breast cancer shows diverse characteristics, including HER2-driven subtypes and viral-driven subtypes. HER2 can be targeted; however, escape of the cancer from targeted therapies remains a problem. In this work we evaluated the capacity of Rlip knockout to prevent breast cancer in genetically engineered mouse models of HER2-driven breast cancer (Erbb2 model) and polyomavirus-driven breast cancer (PyVT model). We found that in Erbb2 mice, Rlip knockout significantly delayed oncogenesis and reduced the expression of genes associated with poor prognosis in patients. In PyVT mice, Rlip knockout did not delay oncogenesis or tumor growth, but Rlip knockdown reduced tumor metastasis to the lung. We conclude that Rlip inhibitors may significantly improve survival in HER2-positive patients, but are unlikely to offer benefits to patients with polyomavirus-associated tumors.

**Abstract:**

We recently reported that loss of one or both alleles of *Ralbp1*, which encodes the stress-protective protein RLIP76 (Rlip), exerts a strong dominant negative effect on both the inherent cancer susceptibility and the chemically inducible cancer susceptibility of mice lacking one or both alleles of the tumor suppressor p53. In this paper, we examined whether congenital Rlip deficiency could prevent genetically-driven breast cancer in two transgenic mouse models: the MMTV-PyVT model, which expresses the polyomavirus middle T antigen (PyVT) under control of the mouse mammary tumor virus promoter (MMTV) and the MMTV-Erbb2 model which expresses MMTV-driven erythroblastic leukemia viral oncogene homolog 2 (Erbb2, HER2/Neu) and frequently acquires p53 mutations. We found that loss of either one or two Rlip alleles had a suppressive effect on carcinogenesis in Erbb2 over-expressing mice. Interestingly, Rlip deficiency did not affect tumor growth but significantly reduced the lung metastatic burden of breast cancer in the viral PyVT model, which does not depend on either Ras or loss of p53. Furthermore, spontaneous tumors of MMTV-PyVT/Rlip+/+ mice showed no regression following Rlip knockdown. Finally, mice lacking one or both Rlip alleles differentially expressed markers for apoptotic signaling, proliferation, angiogenesis, and cell cycling in PyVT and Erbb2 breast tumors. Our results support the efficacy of Rlip depletion in suppressing p53 inactivated cancers, and our findings may yield novel methods for prevention or treatment of cancer in patients with HER2 mutations or tumor HER2 expression.

## 1. Introduction

Breast cancer is the most common cancer in women worldwide and the second most common cancer overall [1]. There were over 2 million new cases of breast cancer in 2018. It is a leading cause of cancer death in developing countries and the second leading cause of cancer death in American women (https://www.wcrf.org, accessed on 3 February 2021). Advanced breast cancer with metastases is considered incurable with currently available chemotherapy regimens [2]. On the molecular level, breast cancer is a heterogeneous disease with diverse genetic and epigenetic alterations, including activation of human epidermal growth factor receptor 2 (HER2, encoded by *ERBB2*), activation of hormone receptors (estrogen receptor and progesterone receptor), *TP53* mutations, and *BRCA* mutations [3]. Additionally, research has pointed toward associations of the Epstein–Barr virus (EBV), herpes simplex virus (HSV), human papilloma virus (HPV), cytomegalovirus (CMV), and Simian Virus 40 (SV40), among others, with human breast cancer [4,5,6,7]. Recently, researchers have identified 3 subgroups of HER2+ patients that clinically behave differently, with one subgroup reported to have a high expression of metastatic markers and very poor 10-year survival [8]. HER2+ patients also have a high prevalence of p53 mutation, with rates reaching 70% [9]. Therefore, prognostic markers that can guide treatment decisions and explain differences in outcome among HER2+ patients are being developed [3]. Based on these studies, it is believed that targeted chemotherapy regimens will be an even more prominent subject in the near future. Treatment strategies differ according to molecular subtype, and management of breast cancer requires a multidisciplinary approach, including locoregional (surgery and radiation therapy) and systemic approaches. Systemic therapies include endocrine therapy for hormone receptor-positive disease, chemotherapy, anti-HER2 therapy for HER2-positive disease, bone stabilizing agents, and poly (ADP-ribose) polymerase (PARP) inhibitors for *BRCA* mutation carriers [10]. Future therapeutic concepts in breast cancer aim at personalization of therapy as well as treatment based on tumor biology and on earlier detection and treatment initiation.

The mouse mammary tumor virus (MMTV) promoter-driven polyomavirus middle T antigen (PyVT) and the MMTV-driven erythroblastic leukemia viral oncogene homolog 2 (Erbb2) mouse models of breast cancer are ideal for oncogenesis studies due to colony stability, predictable tumor growth behavior, and similarity to human neoplasms. These models have broad relevance for understanding the mechanisms of tumor-suppression by p53 and for studying chemopreventive therapies for malignancy. MMTV-PyVT is a model of breast cancer metastasis in which the MMTV-LTR promoter is used to drive the expression of mammary gland-specific polyomavirus middle T-antigen, leading to a rapid development of highly metastatic tumors [11]. MMTV-PyVT mice have been crossbred with other genetically modified mice to study the effects on mammary tumor progression and metastasis of PI3K/Akt signaling [12], colony-stimulating factor-1 (CSF-1) [13], nuclear factor kappa B (NF-_K_B) [14], CD4+ T cells [15], transforming growth factor beta 1 (TGF-β1) [16], plasmin deficiency [17] and MEKK1 [18].

The *ERBB2* (HER2/neu) human oncogene encodes a transmembrane receptor tyrosine kinase glycoprotein which belongs to the epidermal growth factor receptor (EGFR) family. The *ERBB2* gene is amplified or overexpressed in approximately 30% of human breast cancers and in many other cancer types. MMTV-Erbb2 transgenic mice express an activated rat c-neu oncogene and are used to study breast cancer histopathology, oncogenic signaling pathways initiated by aberrant overexpression of HER2 in the mammary epithelium, and interactions between oncogenes and tumor suppressor genes at molecular levels. This mouse model is also useful for antibody or drug investigations aimed at overcoming resistance to trastuzumab or HER2-specific tyrosine kinase inhibitors [19,20,21]. Moreover, HER2+ breast cancer patients, and MMTV-Erbb2 mice have a similar high prevalence of p53 mutations [22].

*TP53* (p53) functions as a stress-responsive, genome protective, tumor suppressor whose functions are lost or altered in a majority of malignancies [23,24,25]. p53 homozygous knockout (p53^−/−^) mice uniformly die of spontaneous malignancies, particularly T-cell lymphoma. Though spontaneous neoplasia can be increased by numerous genetic modifications or carcinogens, no modification of any single gene has itself prevented p53^−/−^ mice from developing spontaneous neoplasia [26]. We recently reported that in stark contrast to p53^−/−^ mice, Rlip^−/−^ mice are highly resistant to carcinogenesis [26]. We also reported that chronic partial depletion of Rlip provides p53^−/−^ mice with remarkable protection from spontaneous malignancy and is associated with the reversion of the aberrant transcriptomic and methylomic patterns of p53^−/−^ mice towards the wild type patterns and with the normalization of the majority of p53-linked cancer suppression pathways [26]. Rlip knockout and depletion effectively suppress or eliminate carcinogenesis in animal models through epigenetic mechanisms that alter the expression of key cancer promoting or preventing genes because of altered haploinsufficiency interactions between Rlip, p53, and other binding partners of these proteins.

Rlip (human gene *RALBP1*, 18p11.22) is a stress-protective [27,28], Ral-regulated [29,30] ATPase of the mercapturic acid pathway that transports glutathione-electrophile conjugates (GS-Es) [27,31,32,33,34,35,36,37,38,39,40,41,42]. It is also an integral component of clathrin-dependent endocytosis (CDE) [37,43,44,45,46,47] and chaperone expression [48,49]. It serves an effector ATPase role in mechanisms that mediate cell cycling, mitochondrial fission, motility, mitosis, and exocytosis [29,30,46,50,51,52], and it mediates resistance to oxidative stress and apoptosis caused by radiation, oxidants, alkylating agents, anthracyclines, and kinase inhibitors. We demonstrated that antibodies against Rlip, antisense oligonucleotides targeted at Rlip (R508), and a small molecule Rlip inhibitor exert potent antineoplastic effects in xenograft models [53,54,55,56,57,58]. R508 treatment in mice reduced Rlip protein expression to 56 ± 12% of controls, reduced blood glucose by 26%, reduced triglycerides by 32%, and reduced cholesterol by 48%, expected pharmacodynamic effects which are consistent with metabolic alterations that are characteristic of Rlip knockout mice [37,59,60].

Rlip depletion or inhibition exerts potent antineoplastic effects in spontaneous and xenograft cancer models. Genetically engineered mice are constructed to model human cancer phenotypes and pathologies, allowing researchers to investigate multiple aspects of cancer. In this study, we used the established genetically engineered MMTV-PyVT and MMTV-Erbb2 mouse models to assess the *in vivo* role of Rlip depletion in mammary carcinogenesis.

The MMTV-Erbb2 transgene is related to the receptor for epidermal growth factor, which is amplified in nearly 30% of human cancers, particularly ductal carcinomas [22]. The MMTV-PyVT transgene is related to an oncogene that activates the non-receptor tyrosine kinase, c-Src, and physically interacts with Taz (*WWTR1*) [61]. A high prevalence of *Trp53* mutations has been reported for the MMTV-Erbb2 model [22], while this has not been reported as a feature of the MMTV-PyVT model. This paper, therefore, describes the effects of genetic RLIP depletion on mammary tumor incidence in MMTV-driven ErbB2 or PyVT expressing transgenic mice. Our results present a potential strategy for treating breast cancer and suggest the utility of Rlip haploinsufficiency in the prevention of breast cancer development and metastasis.

## 2. Materials and Methods

### 2.1. Reagents

Anti -E-cadherin, anti-IκBα, anti-stat2, anti-p42-MAP Kinase, anti-Bcl2, anti-EGFR1, anti-vimentin, anti-p70s6k, anti-CDK4, Cyclin B1, cleaved PARP, Bax, Survivin, PI3K, and anti-beta-actin primary antibodies were purchased from Cell Signaling Technology (Danvers, MA, USA). Rlip-LNA and LNA control antisense (CAS) were purchased from Exiqon (Woburn, MA, USA). Sources of reagents for qRT-PCR, western blot, and genotyping were the same as previously described [62,63,64]. All reagents were of analytical grade.

### 2.2. Ethics Statement

Mice were housed in a vivarium under NIH guidelines, and all animal experiments followed protocols approved by the TTUHSC Institutional Animal Care and Use Committee (IACUC approval no. 18015). All efforts were made to minimize pain and suffering of the animals.

### 2.3. Ralbp1 Knockout Mice

**Figure 1:**
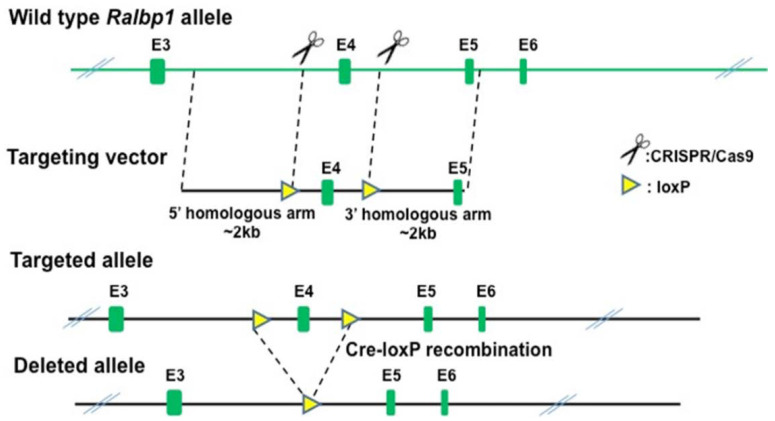
Schematic representation of the wild type and mutant/targeted alleles. This design uses the Cre-loxP system to delete exon 4 of *RALBP1*. The introns occurring between exons 3 and 4 and between exons 4 and 5 are large and insertion of the loxP element will not interfere with mRNA splicing. To minimize the possibility of disruption of EGE-LBX-020 expression, both of the loxP sites were inserted into non-conserved regions. Exon 4 knockout was validated by Southern blot and PCR.

The *Ralbp1* knockout mice were generated by Biocytogen (Beijing, China) using the CRISPR/Cas9-based EGE system. Two single guide RNAs (sgRNAs) were designed to target non-conserved regions of the introns upstream and downstream of exon 4 of *Ralbp1*, using the CRISPR design tool (http://crispr.mit.edu/ accessed on 17 March 2021). The sgRNAs plasmid and Cas9 mRNA were co-injected into C57BL/6 mouse zygotes, and surviving zygotes were transferred into KM albino pseudo-pregnant mice. The genotypes of *Ralbp1* knockout mice were confirmed by PCR amplification and DNA sequencing. The tail tip of each mouse was collected to isolate genomic DNA [65,66]. The targeted region was amplified by PCR (Forward primer 5’-AGTCAGTGCTCTGACCCCCTGAGC-3’, Reverse primer 5’-TTAGAGTGGGCACACCATCAGTCCC-3’). After scanning the gene structure and the size of exons, we found that exon 4 is deleted, resulting in a 307aa (234 native aa plus 73 frame-shift aa) truncated protein which may be subject to non-sense mediated decay (NMD) (Figure 1).

### 2.4. Generation of Rlip-MMTV-Erbb2 and Rlip-MMTV-PyVT GEM Mice

FVB-Tg(MMTV-Erbb2)NK1Mul/J mice (Stock number 005038) and B6.FVB-Tg(MMTV-PyVT)634Mul/LellJ (Stock number 022974) were purchased from the Jackson Laboratory (Bar Harbor, ME, USA). Hemizygous B6.FVB-Tg (MMTV-PyVT)634Mul/LellJ (hereafter PyVT) male mice were mated with *Ralbp1*(C57BL/6J) heterozygous females, which had been backcrossed for eight generations, making the disrupted *Ralbp1* gene and the PyVT transgene available in the C57BL/6 genetic background. All PyVT:Rlip^−/^^−^, PyVT:Rlip^+/−^, and PyVT:Rlip^+/+^ mice used throughout the study were siblings. Similarly, FVB-Tg(MMTV-Erbb2)NK1Mul/J (hereafter Erbb2) male mice were mated with *Ralbp1*(C57BL/6J) heterozygous females, and their offspring were mated with *Ralbp1*(C57BL/6J) heterozygous females (F1) for several generations, making the disrupted *Ralbp1* gene and the Erbb2 transgene available in the C57BL/6 genetic background. To minimize differences in genetic background, we performed all experiments with Erbb2:Rlip^−/−^, Erbb2:Rlip^+/−^, and Erbb2:Rlip^+/+^ mice in the F7 generation.

### 2.5. Genotyping

Chromosomal DNA was isolated from an approximately 2 mm piece of tail tip [65]. Genotyping was performed as suggested by The Jackson Laboratory using recommended primer pairs specific for the PyVT and Erbb2 transgenes. We followed the genotyping protocol for *Ralbp1* optimized by Biocytogen (Beijing, China). Details on primer sequences and PCR cycling and gel pictures of PCR products for each genotype are provided in Figures S1–S3. All pups were weaned at age 21–23 days, genotyped and caged randomly with five mice in each cage. All analyzed mice were female virgins and fed a regular chow.

### 2.6. Tumor Growth and Tissue Processing

Mice were examined twice weekly for mammary tumor onset by palpation for nodules. Tumor volume was assessed weekly by measuring the length, width, and depth of individual tumors with a caliper. The tumor volume was calculated according to the formula: V = L × W × D/2, where L is tumor length, W is tumor width, and D is tumor depth [64]. The individual tumor volumes were summed to give the total tumor volume in each mouse. To minimize final tumor size variation, the mice were euthanized once total tumor burden in each individual mouse reached a preset endpoint size of 2000 mm^3^.

Mice were euthanized at endpoint, and tumor samples were collected and immediately placed on dry ice. Lungs were removed, air-evacuated, and fixed in 10% buffered formalin phosphate before automated paraffin embedding for metastasis quantification. Tumor tissues were cut into ∼5 mm thick slabs followed by H&E staining of embedded tissue by the Pathology Core Facility at TTUHSC [62]. The number of pulmonary metastases was counted under a camera-equipped stereomicroscope.

### 2.7. Dosing of Rlip Antisense Locked Nucleic Acid (Rlip-LNA)

The regression of spontaneous breast cancer tumors by Rlip-LNA treatment was assessed in the PyVT transgenic mouse model. This antisense treatment has been shown to suppress the growth of murine xenografts of MD-MB-231 and MCF7 cells [62] and targets a region that is identical in the human *RALBP1* and mouse *Ralbp1* mRNA sequences. When tumors were ~50–100 mm^3^ in volume, mice started receiving treatments with thrice weekly i.p. injections of 0.2 mL PBS containing control scrambled antisense (CAS) or Rlip-LNA (0.2 mg/0.2 mL). Mice were inspected daily and injections were continued until humane euthanasia was necessary due to pre-specified signs of debility, distress, or tumors.

### 2.8. Determination of mRNA Levels by Real-Time Polymerase Chain Reaction

Total RNA was isolated from tumor tissue samples obtained from PyVT:Rlip^−/−^, PyVT:Rlip^+/−^, PyVT:Rlip^+/+^, Erbb2:Rlip^−/−^, Erbb2:Rlip^+/−^, and Erbb2:Rlip^+/+^ mice by the guanidinium thiocyanate method, using TRIzol reagent from Thermo Fisher Scientific (Waltham, MA, USA). Complementary DNA was prepared using the SuperScript IV VILO Master Mix (Thermo Fisher Scientific, Waltham, MA, USA. In brief, genomic DNA was removed from 2.5 µg of RNA using ezDNase enzyme provided with the kit. cDNA was then prepared according to the manufacturer’s instructions by mixing RNA with SuperScript IV VILO Master Mix and incubated at 25 °C for 10 min followed by incubation at 50 °C for 10 min; the enzyme was inactivated by bringing the temperature to 85 °C for 5 min. Real-time polymerase chain reaction (qRT-PCR) was performed in replicate on a 7900HT Fast Real-Time PCR System (Life Technologies Corporation, Grand Island, New York, NY, USA) with PowerUp SYBR Green Master Mix (Thermo Fisher) in a total reaction volume of 20 µL containing 0.3 µM gene-specific primers. The cycling protocol was an initial denaturation at 95 °C, followed by 40 cycles of denaturation at 95 °C and annealing/extension at 60 °C. The ribosomal protein S3 (RPS3) transcript was used as a reference for normalization of mRNA levels. Gene expression levels were normalized for each individual animal by the ΔΔCt method. For both the Erbb2 and PyVT model systems, mRNA expression of the Rlip^+/−^ and Rlip^−/−^ genotypes was normalized to the corresponding Rlip^+/+^ genotype. Primers for each gene are detailed in Table 1. Results are presented as mean and standard deviation (*n* = 3).

### 2.9. Assessment of Angiogenesis, Proliferation, and Apoptotic Signaling

Tissue lysates were prepared from tumors and a full Western blot panel was performed for all mice that had detectable tumors at endpoint, resulting in *n* ≥ 3 for all treatment cohorts. Tumor protein was prepared using 2× Laemmli Sample Buffer (Bio-Rad, Hercules, CA, USA) and was loaded on 4–12% Bis-Tris gels (45 μg protein/lane), with 1× MES gel running buffer. Proteins were transferred to nitrocellulose membrane, and blocking was done in Pierce Clear Milk Blocking Buffer (Thermo Fisher) with 0.1% TWEEN 20 for 1 h at room temperature. Membranes were probed with mouse or rabbit mono/polyclonal primary antibodies for anti-E-cadherin, anti-IκBα, anti-stat2, anti-p42-MAP Kinase, anti-Bcl2, anti-vimentin, anti-p70s6k, anti-cyclin B1, anti-cleaved PARP1, anti-Bax, anti-survivin, anti-PI3 Kinase Class III, and anti-CDK4. All antibodies were diluted to 1:1000 in 1× Clear Milk + 0.1%TWEEN 20. After overnight incubation at 4 °C with gentle rocking, membranes were washed five times (5 min each at room temperature) with Tris-buffered saline-TWEEN 20 (TBST; 20 mM Tris-HCl (pH 7.6), 137 mM NaCl, and 0.2% (*v/v*) TWEEN 20), and incubated with horseradish-peroxidase-coupled anti-IgG (secondary antibody, dilution 1:2000) in 1× Clear Milk + 0.1% TWEEN 20 for 1 h at room temperature. Bands were visualized using SuperSignal West Pico PLUS Chemiluminescent Substrate (Thermo Fisher) following the manufacturer’s instructions. For the loading control, at the end of the experiments, nitrocellulose membranes were stripped with Restore Western Blot Stripping Buffer (Thermo Fisher) and re-probed with anti-beta-actin antibody (1:1000 dilution, Santa Cruz Biotechnology). Bands were visualized using an ImageQuant LAS4000 (GE Healthcare Life Sciences, PA, USA).

### 2.10. Statistical Analysis

All analyses were performed using Prism 9.0 for Windows (GraphPad, San Diego, CA, USA). Results are reported as mean ± SD. Data were analyzed for significance by ANOVA with the post-hoc Tukey–Kramer or Newman–Keuls test; survival curves were analyzed using the logrank test, and *p*-values less than 0.05 were considered significant.

## 3. Results

### 3.1. Effects of Rlip Depletion on Spontaneous Tumor Development

The genetic complexities of different breast cancer subtypes drive initiation and progression differently, and genetically engineered mouse models (GEMMs) can serve as tools to study the diverse molecular aspects of this disease. We previously reported that genetically- or chemically-induced Rlip depletion or inhibition strongly inhibited spontaneous carcinogenesis, xenograft tumor growth, and chemically-induced carcinogenesis in mice [26,67]. We report here that genetic depletion of Rlip in MMTV-Erbb2 (C57BL/6) mice significantly delays spontaneous carcinogenesis, but MMTV-PyVT (C57BL/6) mice do not show significant effects with genetic Rlip depletion.

**Figure 2:**
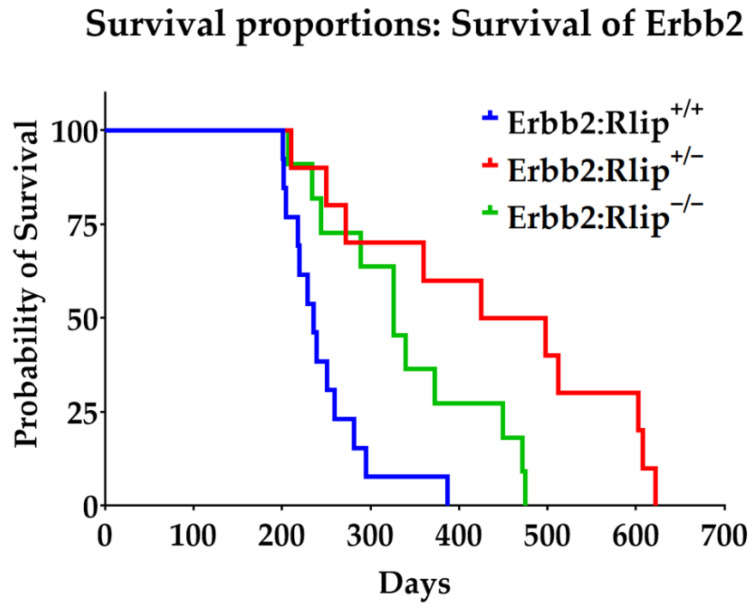
Rlip deficiency prevents spontaneous carcinogenesis in the MMTV-Erbb2 mouse model. Overall survival curves for mice of the indicated genotypes are shown. No treatment was given, and the mice were euthanized when tumor volume reached 2000 mm^3^. Results are reported as mean ± SD (Erbb2:Rlip^+/+^ (*n* = 13); Erbb2:Rlip^−/−^ (*n* = 10) and Erbb2:Rlip^+/−^ (*n* = 11)).

**Figure 3:**
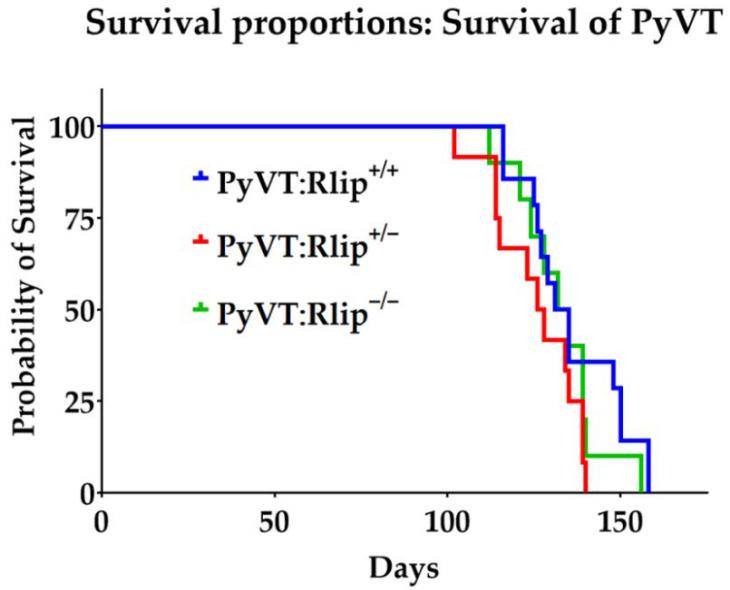
Rlip deficiency does not prevent spontaneous carcinogenesis in viral MMTV-PyVT mouse model. Overall survival curves for mice of the indicated genotypes are shown. No treatment was given, and the mice were euthanized when tumor volume reached 2000 mm^3^. Results are reported as mean ± SD (PyVT:Rlip^+/+^ (*n* = 14); PyVT:Rlip^−/−^ (*n* = 12) and PyVT:Rlip^+/−^ (*n* = 10)).

Median survival of Erbb2:Rlip^+/+^, Erbb2:Rlip^−/−^ and Erbb2:Rlip^+/−^ mice was 33.7, 46.5, and 65.9 weeks, respectively (Figure 2 and Table S1). On the other hand, the median survival of PyVT:Rlip^+/+^, PyVT:Rlip^−/−^ and PyVT:Rlip^+/−^ mice was 19, 19, and 18 weeks, respectively (Figure 3 and Table S2). Comparing survival curves for the Erbb2:Rlip^+/+^, Erbb2:Rlip^−/−^, and Erbb2:Rlip^+/−^ genotypes via logrank (Mantel–Cox) and Gehan–Breslow–Wilcoxon tests (GraphPad Prism version 9, GraphPad Software Inc., San Diego, CA, USA) showed a significant difference in survival rates between the Erbb2:Rlip^+/+^ mice and the Erbb2:Rlip^+/−^ and Erbb2:Rlip^−/−^ mice (*p* < 0.002, Table S1). Survival curve comparisons of the PyVT:Rlip^+/+^, PyVT:Rlip^−/−^ and PyVT:Rlip^+/−^ genotypes using the same parameters did not show any significant changes (Table S2). The tumor volumes at endpoint are plotted in Figure S4. All mice developed multi-focal mammary tumors along the lacteal glands. Some of them were often found under the arms, neck, sides of body or on the upper back. Most tumors were solid lumps with malignant morphologic features. Thus, Rlip deficiency exerted a strong dominant effect on the spontaneous malignancy phenotype of Erbb2 mice but not of PyVT mice.

**Figure 4:**
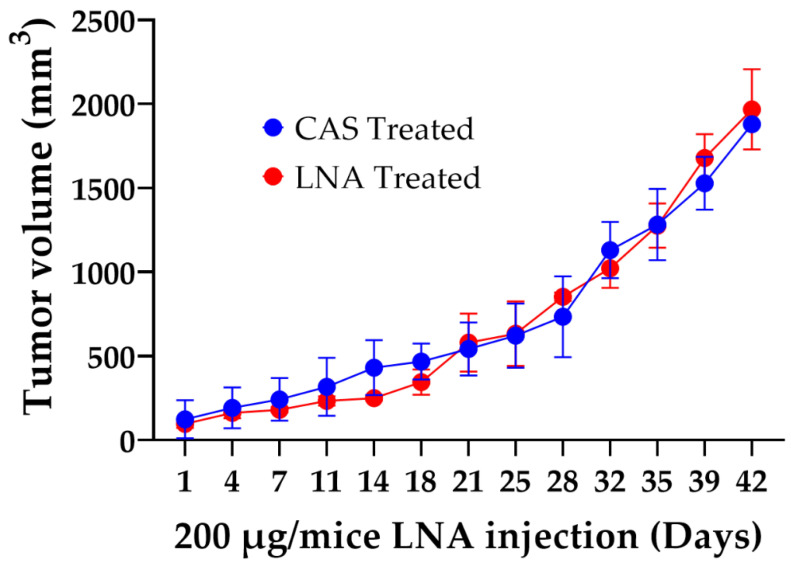
Effect of Rlip-LNA on growth rate of PyVT tumors. Tumor growth was monitored twice weekly, and the tumor sizes were determined using a caliper as described in materials and methods. The graph shows the growth of tumors in the control anti-sense (CAS) and treated (Rlip-LNA) groups. Results are reported as mean ± SD (*n* = 5 each group).

In vivo Rlip depletion against several cancer types results in regression or slower growth of tumors and improved survival [26,58,62,63]. Surprisingly, genetic depletion of Rlip in the MMTV-PyVT GEMM showed no effect. To further validate this result, we conducted studies on the antineoplastic efficacy of Rlip depletion by antisense (Rlip-LNA) in PyVT:Rlip^+/+^ mice with breast cancer tumors. Treatment with 0.2 mg (~8 mg/kg body weight) Rlip-LNA was initiated as described in Materials and Methods when tumor volume was ~50–100 mm^3^. Administration of Rlip-LNA had no significant effect on tumor progression in this model (Figure 4). This further confirms that Rlip depletion does not slow tumor growth or improve survival in the PyVT viral carcinogenesis model.

### 3.2. Congenital Rlip Deficiency Prevents Lung Metastasis

**Figure 5:**
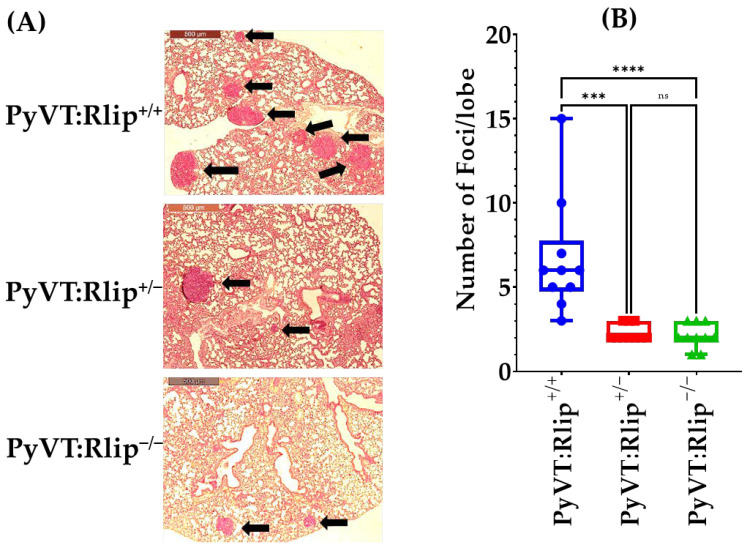
Rlip knockout reduced tumor lung metastases in PyVT:Rlip^−/−^ and PyVT:Rlip^+/−^ mice. (**A**) Low-magnification (40×) H&E-stained images of lungs showing tumor metastases (arrows) in approximately 140–150 day old female PyVT:Rlip^+/+^, PyVT:Rlip^−/−^, and PyVT:Rlip^+/−^ mice. (**B**) Number of metastatic foci in lung of PyVT:Rlip^+/+^, PyVT:Rlip^−/−,^ and PyVT:Rlip^+/−^ mice. Foci were counted in a blinded fashion and values are presented as means ± SD (*n* = 5 each group); ns = not significant; *** = *p* < 0.001 and **** = *p* < 0.0001.

It is well documented that spontaneous tumors in MMTV-PyVT female mice metastasize to the lung [68,69,70]. We have previously reported that Rlip depletion prevents metastasis to the lung in chemical carcinogenesis and p53-mutated mouse models [71]. Therefore, we tested whether congenital depletion of Rlip would prevent metastasis of mammary tumors to the lung. Interestingly, we found that there was a significant difference in the incidence of lung metastasis between PyVT:Rlip^+/+^, PyVT:Rlip^−/−^, and PyVT:Rlip^+/−^ mice. We counted significantly higher numbers of metastatic tumors in PyVT:Rlip^+/+^ (6.7 ± 3.5) compared to PyVT:Rlip^−/−^ (2.1 ± 0.74) and PyVT:Rlip^+/−^ (2.3 ± 0.48) mice (Figure 5). Additionally, it was evident that the cross sectional areas of the lung metastases were smaller in the PyVT:Rlip^−/−^ mice compared to the PyVT:Rlip^+/+^ mice. Gross necropsy at termination found no metastatic lesions in other organs.

### 3.3. Effect of Rlip Depletion on Key Tumor Proteins Involved in Progression and Signaling

Understanding the biology of this malignant disease is a prerequisite for selecting an appropriate treatment. We have previously demonstrated that Rlip depletion in xenograft and chemical carcinogenesis models affects the expression of key proteins involved in tumor progression, but this has not been studied in GEMMs.

**Figure 6:**
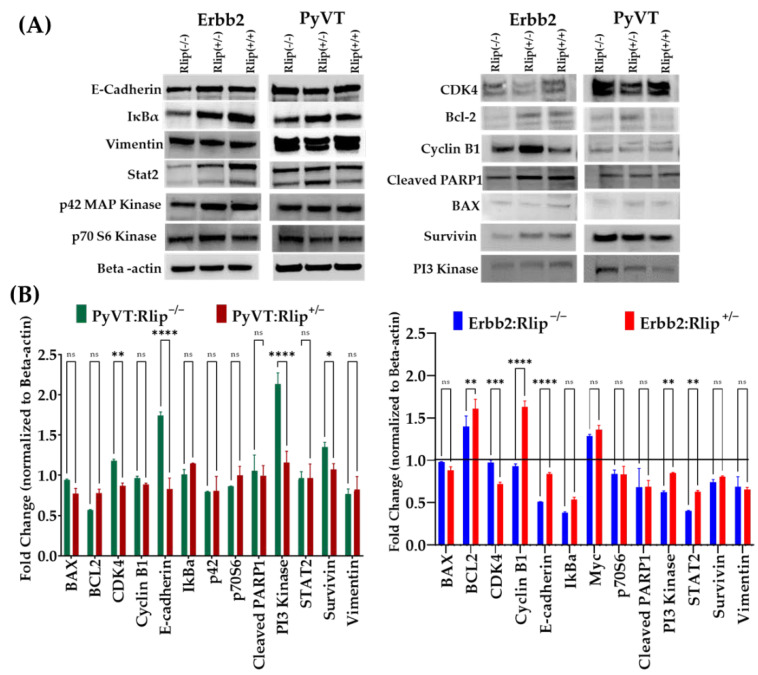
(**A**) Effect of genetic Rlip knockout on the levels of cell survival, proliferation, apoptosis, and differentiation marker proteins in PyVT and Erbb2 breast cancer tumors. Western blots showing apoptosis, cell survival, proliferation, and differentiation marker proteins in lysates of tumors collected at study endpoints from Erbb2:Rlip^+/+^, Erbb2:Rlip^−/−^, Erbb2:Rlip^+/−^, PyVT:Rlip^+/+^, PyVT:Rlip^−/−^, and PyVT:Rlip^+/−^ mice. Representative Western blots are shown in the figure (*n* = 3 tumors from each genotype). β-actin was used as a loading control. Detailed information about Western Blots can be found in the supplementary materials. (**B**) Effects of Rlip depletion on protein expression of markers of intracellular signaling in PyVT and Erbb2 mammary tumors. Western blot densitometry of tumor tissue lysates from PyVT:Rlip^+/+^, PyVT:Rlip^+/−^, PyVT:Rlip^−/−^, Erbb2:Rlip^+/+^, Erbb2:Rlip^+/−^, and Erbb2:Rlip^−/−^ mice. The bar diagrams represent the fold change in protein levels in Rlip knockout genotypes (Rlip^+/−^ and Rlip^−/−^) as compared to Rlip^+/+^ genotypes (defined as = 1, indicated by the solid line). Values are shown as mean +/− SD (*n* = 3 tumors from each genotype; ns = not significant, * = *p* < 0.01; ** = *p* < 0.005; *** = *p* < 0.001 and **** = *p* < 0.0001). β-actin was used as a loading control.

Western blot analysis indicated that several cancer hallmark pathways were suppressed following genetic Rlip depletion in tumors obtained from PyVT and Erbb2 GEMMs (Figure 6A,B and Figures S5–S17). Expression of anti-apoptotic BCL2, p42 MAP kinase, and epithelial marker E-cadherin was reduced in Erbb2:Rlip^−/−^ but remained unaffected in tumors from the PyVT genotypes. Rlip depletion also decreased expression of STAT2 (signal transducer and activator of transcription 2) and CDK4, a protein involved in proliferation and cell cycle progression, in the Erbb2:Rlip^−/−^ and Erbb2:Rlip^+/−^ tumors, but these proteins were unaffected by loss of Rlip in the PyVT model. These results largely correlate with the finding that the targeting of Rlip inhibits breast cancer oncogenesis in Erbb2 GEMMs, but not in PyVT GEMMs.

### 3.4. mRNA Expression of Key Breast Cancer-Related Genes in Tumor Biopsies

**Figure 7:**
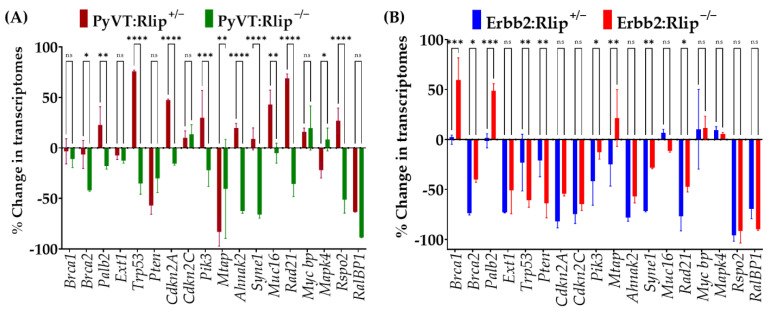
Effects of Rlip depletion on relative levels of mRNA transcripts encoding key genes associated with breast cancer in (**A**) PyVT and (**B**) Erbb2 mammary tumors. Tumor transcript levels were determined by qRT-PCR and calculated as described in Materials and Methods. Transcript levels of all genotypes were normalized to the S3 ribosomal protein transcript. Results are shown as % change in transcript levels of heterozygous or homozygous Rlip knockout genotypes relative to the corresponding Rlip^+/+^ genotypes. The values are shown as means ± SD (*n* = 3 tumors from each genotype; ns = not significant, * = *p* < 0.01; ** = *p* < 0.005; *** = *p* < 0.001 and **** = *p* < 0.0001).

The mRNA transcript levels of breast cancer-specific cell surface genes, tumor suppresser genes, cell growth/proliferation-related genes, and oncogenes are often examined to understand the actions of antineoplastic agents and to predict clinical efficacy. Since congenital Rlip knockout has differential effects on PyVT-driven and Erbb2-driven mammary carcinogenesis, we examined the expression of a number of genes related to breast cancer by qRT-PCR in breast tumors obtained from all six genotypes. The selected genes (Figure 7) fell into functionally distinct classes that represent multiple events of oncogenic transformation, such as oncogene activation and tumor suppressor inhibition. Transcript levels of a number of these genes were differentially increased or decreased in tumors obtained from Rlip^+/−^ or Rlip^−/−^ PyVT or Erbb2 mice, relative to Rlip^+/+^ PyVT or Erbb2 mice (Figure 7A,B).

In PyVT mouse tumors (Figure 7A), transcript levels of *Brca1*, *Palb2*, *Ext1*, *Mycbp*, *Mapk4*, *Cdkn2c*, and *Pik3ca* were least affected (0–25%) by the loss of one or both copies of Rlip, and *Brca2*, *Syne1*, *Rspo2* were moderately decreased with homozygous Rlip knockout, but were little affected by the loss of one copy of Rlip. On the other hand, transcript levels of *Pten* and *Mtap* were decreased by 30–80% in tumors from both hetero- and homozygous Rlip knockout mice, and *Brca2*, *Syne1*, *Rspo2*, *Ahnak2*, *Rad21* were reduced by 35–65% by the loss of both copies of Rlip in PyVT mice. In PyVT mouse tumors, transcript levels of *Trp53* registered opposite effects with the Rlip^+/−^ genotype (a 75% increase) compared to the Rlip^−/−^ genotype (a 35% decrease). In Erbb2 mouse tumors (Figure 7B), transcript levels of *Mapk4*, *Mycbp*, *Muc16*, *Mtap* were least affected (0–25%) by the loss of one or both copies of Rlip, and *Brca1* and *Palb2* were little affected by the loss of one copy of Rlip but were increased by 50–60% by the loss of both copies of Rlip. On the other hand, transcript levels of *Brca2*, *Ext1*, *Trp53*, *Pten*, *Cdkn2a, Cdkn2c*, *Pik3ca*, *Ahnak2*, *Syne1*, *Rad21*, and *Rspo2* were decreased by 30–95% by loss of one copy or both copies of Rlip.

These results show that the majority of key genes associated with breast cancer progression and proliferation were downregulated in Rlip-depleted Erbb2 mouse tumors. On the other hand, with the exception of *Pten* and *Mtap*, the transcript levels of the Rlip knockout PyVT genotypes showed no consistent up- or downregulation and, in some cases, the changes were less than 25% relative to the Rlip^+/+^ PyVT genotype. These results suggest that Rlip depletion affects gene expression differentially in these two cancer types, and thus, the observed patterns of tumor growth and prevention.

### 3.5. Computational Modeling of Rlip-p53 Complex

We have previously found that p53 and Rlip proteins interact [72], and that knockdown of Rlip prevents cancer in p53 knockout mice [26]. The sequence alignment among Rlip/PyVT/SV40 was analyzed by using Clustal Omega [73]. The three-dimensional structural alignment was performed using PyMOL (The PyMOL Molecular Graphics System, Version 1.2r3pre, Schrödinger, LLC, New York City, NY, USA). To explore the possible binding mode of the Rlip-p53 complex, ZDOCK software [74] was used to generate 5000 complex models. The structure of Rlip was adapted from its NMR structure (PDB id 2mbg) [75], and that of p53 was extracted from the structure of the complex of oncoprotein SV40 large T antigen and p53 (PDB id 2h1l) [76]. The best model with largest interaction score was picked out by using our in-house developed LiAn (Legion Interfaces Analysis) program [77].

**Figure 8:**
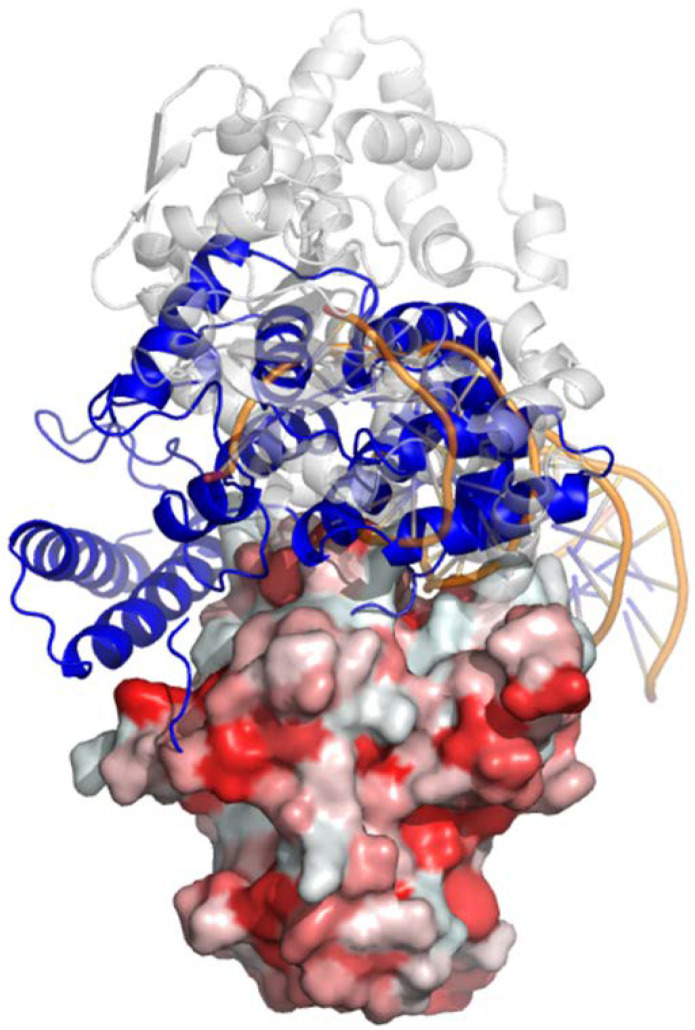
Predicted model of Rlip (blue-colored) interacting with p53. p53 is colored as red hydrophobic surface. The LT_SV40 (grey-colored) and DNA (orange-colored) that bind to p53 are also superimposed.

**Figure 9:**
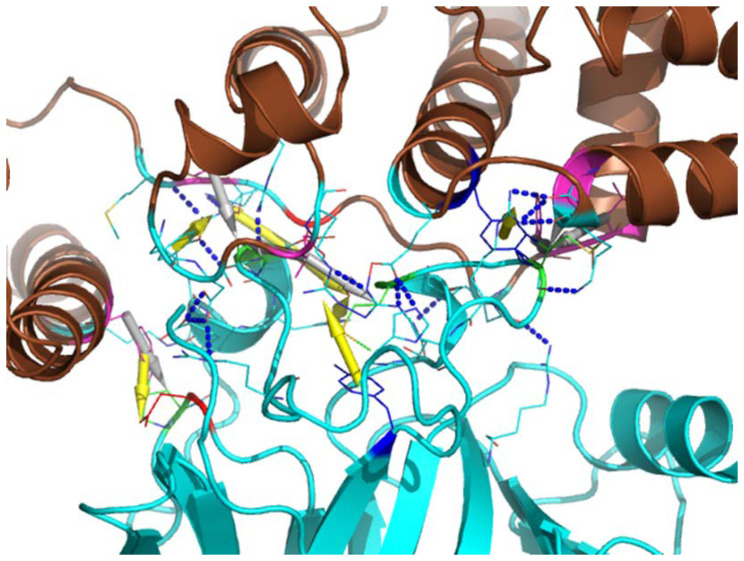
Predicted interface of Rlip and p53 complex. The hydrogen bonds are shown as blue dots, while the hydrophobic interactions are displayed as grey arrows and π-interactions are depicted as yellow arrows.

As depicted in Figure 8, the best model shows that Rlip may bind the DNA-binding region (PDB id 1tsr [78]) of p53, an interface which overlaps with the interface used by SV40 (PDB id 2h1l). As shown in Figure 9, 17 pairs of hydrogen bonds or salt-bridges are formed between the two proteins, i.e., R174(p53) –D205(Rlip), R181–M377, R181–Q375, R248–L307, R248–K308, R248–L307, R249–E312, R249–R208, R273–N370, N239–N370, E180–D205, H168–E312, H178–R214, H179–R403, M246–K308, P177–Y204, and S241–N370. Besides the strong hydrophilic interactions on the protein–protein interaction surface, 7 pairs of hydrophobic interactions are also presented to enhance the complex binding, i.e., P191(p53) –F407(Rlip), P177–I207, M246–L307, M246–F368, M246–L367, M243–I207 and P177–V376. Furthermore, the protein complex structure is also strengthened by several π-cation interactions, such as K374(p53) –H179(Rlip), R248–Y315, Q192–F407, R181–Y204 and R208–Y163.

## 4. Discussion

Rlip is a stress-protective, anti-apoptotic mercapturic acid pathway transporter protein in the Ral/Rac/Rho cancer-signaling pathways which also facilitates the efflux of toxic ω-6 fatty acid metabolites and drugs from cells to increase cancer cell survival [26,58,62]. We have previously demonstrated that Rlip depletion prevents or treats chemically induced, xenograft and spontaneous cancers in mouse models and has existential importance for cancer cell formation and survival [26,58,62,79,80]. The functions of the tumor suppressor p53 are lost or altered in a majority of breast cancers. *Trp53*^−/−^ mice uniformly die of spontaneous malignancy [26]. In stark contrast to *Trp53*^−/−^ mice, mice with hetero- or homozygous loss of Rlip are highly cancer resistant, even when challenged with the most potent known chemical carcinogens, such as benzo[a]pyrene or dimethylbenzanthracene [26]. Our recent studies showed for the first time that pharmacologically or genetically induced deficiency of Rlip protein ‘switched off’ spontaneous carcinogenesis in p53-null mice, essentially bypassing the requirement of p53 to suppress cancer [26]. We found that cancer-free survival was associated with the prevention of age-acquired promoter CpG island methylation abnormalities in the p53-null mice. That anti-cancer effect of Rlip depletion is corroborated by this work and by prior studies to elucidate the activity [81,82], identity [30,32,83], functions [50,84,85,86] and potential clinical applications [41,58,63] of Rlip. Our discovery that Rlip haploinsufficiency exerts a dominant negative effect on spontaneous carcinogenesis in p53-deficient mice has shifted the paradigm of understanding p53 function by introducing previously unknown protein interactions that interrupt the accumulation of epigenetic alterations associated with p53 deficiency. Because Rlip protein protects *TP53* deficient cancer cells [26], here we tested whether Rlip depletion would prevent the inevitable development of spontaneous cancer in GEM mouse models of breast cancer.

The disruption of the *Ralpb1* gene through the CRISPR/Cas9-based EGE system was successful, as shown by Southern blotting and, independently, by PCR (Figure S1). While only one coding exon of the gene was deleted, essentially no *Ralbp1* mRNA was detected by Northern blotting in any of the tissues examined. Even if a low-level expression of mRNA were to occur, the deletion of a structurally essential part of the protein sequence would prevent the synthesis of a functional Rlip protein. This was indeed the case, as determined by western blotting. Mice heterozygous with respect to the intact *Ralbp1* allele expressed intermediate levels of mRNA and protein.

Switching off spontaneous and chemical carcinogenesis in p53^−/−^ mice is especially significant because this was possible by depletion of Rlip to only a hemizygous level, a defining characteristic of haploinsufficiency phenomena. Here, we showed that genetic depletion of Rlip significantly delayed spontaneous tumor growth in MMTV-Erbb2 GEMMs, but not in MMTV-PyVT GEMMs. The results obtained from the MMTV-PyVT GEMMs were further validated by Rlip depletion using Rlip-LNA treatment in tumor-bearing PyVT:Rlip^+/+^ mice. Together these results suggest that Rlip has minimal or no therapeutic effects in the viral PyVT mouse model of breast cancer, but that targeting Rlip will delay/treat HER2/ neu-positive breast cancer. This onco-preventive effect of Rlip knockdown in MMTV-Erbb2 mice, which frequently develop p53-mutated tumors, is consistent with our prior finding that Rlip depletion is onco-preventive against mice with *Trp53* deletion mutations [26].

MMTV-PyVT mice are known to readily develop pulmonary metastases [68,69,70]. We found reduced lung metastases in the PyVT:Rlip^+/−^ and PyVT:Rlip^−/−^ mice relative to PyVT:Rlip^+/+^ mice. The cause of this effect is not clear. Tumor metastasis involves tumor cell migration, invasion, interactions with the extracellular matrix, and survival at a distant site from the primary tumor mass, as well as tumor cell proliferation. The effect of Rlip depletion here may be related to reduced cancer cell migration or invasion resulting from altered cytoskeletal behavior, or may be due to altered extracellular microenvironment of the lung preventing efficient invasion of lung tissue. Indeed, we have previously found that murine cancer cell lines failed to proliferate, migrate, and neovascularize when allografted into in Rlip^−/−^ knockout mice [87], suggesting that tissue microenvironments can also contribute to the anticancer effects of Rlip depletion.

The significant delay in tumor formation in the Erbb2 mouse model by Rlip depletion has significant implications of its own. *ERBB2*/HER2 expression, occurring in 10–34% of breast cancer cases, is an important predictor of patient outcome. Association with aggressive disease and poor clinical outcome, together with the high prevalence, has made ERBB2 an attractive target for therapy. Herceptin, an antibody-based therapy directed against the extracellular domain of ERBB2, has shown limited therapeutic efficacy against ERBB2-positive breast cancer [88]. A significant fraction of patients do not benefit from Herceptin treatment and develop type II chemotherapy-related cardiac dysfunction, indicating that other factors beyond ERBB2 itself must influence therapeutic response in ERBB2-positive tumors [89]. Therefore, combination therapies targeting Rlip and ERBB2 may benefit patients suffering from ERBB2-positive metastatic breast cancer. PyVT and Simian virus 40 (LT_SV40) are used to identify regulatory proteins and to differentiate mechanisms by which these proteins exert their biological effects. Analysis suggests that these two proteins share sequence identity and have conserved molecular chaperone DnaJ domains (Supplemental Data S1). Exploring how DNA tumor viruses induce cancer, researchers have concentrated on a small DNA tumor polyomavirus, Simian Virus 40 (SV40), which encodes two tumor antigens, the small t antigen and large T antigen. Identifying interactions between tumor antigens, cellular proteins, and antisera, several studies discovered p53 protein in a variety of cancer cells. Ours and others’ analyses [76] suggest that p53 interacts with the SV40 large T antigen (which shares a great deal of sequence similarity with PyVT with overall sequence identity of 13.5%); thus, it is possible that p53 may interact with PyVT as well. Rlip shares a small degree of sequence and structural similarity with SV40 (with overall sequence identity of 15.2% and RMSD = 5.5Å from three-dimensional structural alignment). We also built a computational model for the Rlip/p53 complex, which shows that Rlip may bind the DNA-binding region of p53 through strong networks of hydrogen bonds, salt-bridges, hydrophobic interactions, and π-cation interactions. Therefore, we hypothesize that depletion of Rlip in PyVT GEM models does not prevent tumorigenesis because p53 replaces Rlip’s function in this model.

The gene expression profile of breast cancer-associated genes is different in Rlip^+/−^ and Rlip^−/−^ PyVT and Erbb2 mouse tumors relative to Rlip^+/+^ PyVT and Erbb2 tumors. We selected genes that predict poor prognosis in patients with advanced breast cancer. Most of the transcript levels such as that of *Palb2* (Partner and Localizer of *Brca2*), *Brca1* and *Brca2* (DNA repair), *Ext1*, *Cdkn2c* (cyclin-dependent kinase inhibitor 2C), *Mycbp* (Myc binding protein), and *Mapk4* (mitogen-activated protein kinase 4) were little affected by loss of one or two copies of Rlip in the PyVT GEMM. Transcript levels of *Trp53*, *Cdkn2a*, *Pik3ca*, *Ahnak2* (Ahnak nucleoprotein 2), *Rspo2* (R-spondin 2), *Syne1* (spectrin repeat containing nuclear envelope protein 1), *Muc16* (mucin 16, cell surface associated) and *Rad21* were upregulated in PyVT/Rlip^+/−^ mice, but were downregulated in PyVT/ Rlip^−/−^ mice. Only *Pten* and *Mtap* (methylthioadenosine phosphorylase) were downregulated by depletion of Rlip in the PyVT GEMM. On the other hand, loss of one or two copies of Rlip downregulated most of the transcripts in tumors obtained from Erbb2 GEM mice, except *Mtap*, *Muc16*, *Mycbp*, *Mapk4*, *Brca1* and *Palb2*. These expression studies largely correlate with the survival studies showing that Rlip depletion delays carcinogenesis in Erbb2 mice but not in PyVT mice. In humans, ERBB2-positive breast cancers frequently harbor *TP53* mutations, often resulting in inactivation and accumulation of p53 protein in the tumor cells. Interestingly, Rlip knockout in the Erbb2 mouse models reduced *Trp53* transcript levels in tumors by ~50%. We have previously demonstrated that Rlip depletion in p53 mutant mice prevents chemical and spontaneous carcinogenesis via complex haploinsufficiency interactions involving Rlip and p53. Depletion of accumulated mutant p53 may enhance the anticancer activity of Rlip depletion. If indeed this phenomenon is supported by future studies, the differential expression of tumor suppressor genes in normal versus tumor tissues would allow for the selective targeting of p53 in tumors.

Western blot analyses of tumor tissue lysates from Rlip^+/+^, Rlip^+/−^ and Rlip^−/−^ genotypes of the Erbb2 and PyVT GEM models revealed significant differences in the patterns of signaling proteins. In PyVT GEMs most of the protein expression levels were unchanged by genetic Rlip depletion except for BCL2, which was downregulated, and PI3 Kinase, E cadherin, and Survivin which were upregulated in PyVT:Rlip^−/−^ mice. The role of BCL2, an anti-apoptotic protein, is dependent on the estrogen receptor (ER) status, which may explain the ineffectiveness of Rlip depletion in the PyVT viral cancer model. Increased levels of PI3 Kinase and Survivin (a member of the inhibitor of apoptosis (IAP) protein family) in tumors may also contribute to the poor response to Rlip depletion in PyVT GEMs. Rlip depletion in the Erbb2 GEM model reduced E-cadherin, an important protein in epithelial mesenchymal transition (EMT), and reduced the expression of most of the key signaling molecules for EMT, including IκBα (nuclear factor of kappa light polypeptide gene enhancer in B-cells inhibitor, alpha), p42, P70s6, Survivin, and STAT2. Levels of Bcl2 were decreased and Cyclin B1was upregulated by Rlip knockout. Differential expression of these signaling and regulatory proteins in the PyVT and Erbb2 models after Rlip depletion correlates with the observed differences in survival and tumorigenesis patterns, and may well contribute to them.

## 5. Conclusions

In a mouse model engineered to mimic HER2-positive breast cancers, Rlip knockout significantly counteracted oncogenesis and reduced the expression of genes associated with poor prognosis in patients. From this result, we conclude that Rlip inhibition strategies may offer therapeutic benefits to HER2-positive breast cancer patients or to patients bearing germline or tumor HER2 mutations. Rlip inhibition is a potential advancement in individualized therapeutic approaches specifically targeting the tumor biology of a high-risk molecular profile of breast cancer.

In the PyVT mouse model, Rlip knockout did not delay oncogenesis or tumor growth, and Rlip knockdown did not regress PyVT-driven mammary tumors, suggesting that Rlip inhibition strategies may be inappropriate in the prevention or treatment of breast cancer in patients with SV40 infections or with tumors positive for polyomavirus DNA, respectively. Interestingly, Rlip knockout reduced lung metastases in PyVT mice, but we cannot conclude whether this observation was due to reduced metastatic potential of the cancer cells or to an altered tissue microenvironment resulting from Rlip knockout. The role of Rlip inhibition in addressing lung metastasis in this cancer needs further investigation and understanding.

## Figures and Tables

**Table 1:** qRT-PCR oligonucleotide primers.

Gene	Forward Primer	Reverse Primer
*Brca1*	CGAATCTGAGTCCCCTAAAGAGC	AAGCAACTTGACCTTGGGGTA
*Brca2*	TGGTAGATGTTGCTAGTCCGC	ACCACTGGCTTTTCTCGTTGT
*Palb2*	GGGAAGCCCCTCAGCTATG	CGAGCAAGTGTCCTGCTGTAT
*Ext1*	TGGAGGCGTGCAGTTTAGG	GAAGCGGGGCCAGAAATGA
*Trp53*	CTCTCCCCCGCAAAAGAAAAA	CGGAACATCTCGAAGCGTTTA
*Pten*	CCTTTTGAAGACCATAACCCACC	GAATTGCTGCAACATGATTGTCA
*Cdkn2A*	CGCAGGTTCTTGGTCACTGT	TGTTCACGAAAGCCAGAGCG
*Cdkn2C*	GGGGACCTAGAGCAACTTACT	AAATTGGGATTAGCACCTCTGAG
*Pik3CA*	CCACGACCATCTTCGGGTG	GGGGAGTAAACATTCCACTAGGA
*Mtap*	ACGGCGGTGAAGATTGGAATA	ATGGCTTGCCGAATGGAGTAT
*Ahnak2*	CCACCCCAACTGGGACTTTG	CACTCCCCTGTAACTTGCCTG
*Syne1*	AGACTGCGACTGCGATGTC	CTGTGCTGTGTTTCTCGATGT
*Muc16*	ACTTCTCACCATTGGCTCGG	AACTGGGTACCATTGTGCGT
*Rad21*	ATGTTCTACGCACATTTTGTCCT	CATGGGCTTTGGTTAGCTTCT
*Mycbp*	GCTGGACACGCTGACGAAA	TCTAGGCGAAGCAGCTCTATTT
*Mapk4*	GCCAGCGTCTACGGGTATG	GCGTCACTCAGAACGATCTTCTT
*Rspo2*	ACGCAATAAGCGAGCTAGTTATG	ACATCGGCTGCAACCATTGT

## Data Availability

The data presented in this study are available in the article and supplementary material.

## Supplementary Materials

The following are available online at https://www.mdpi.com/article/10.3390/cancers13133329/s1, Figure S1: Genotyping details for Ralpb1 wild type and knockout genes as optimized by Biocytogen, Figure S2: Genotyping details for the PyVT transgene as optimized by Jackson Laboratories, Figure S3: Genotyping details for the Erbb2 transgene as optimized by Jackson Laboratories, Figure S4: Tumor sizes at endpoint for PyVT and ERBB2 mice with wild type, heterozygous knockout, or homozygous knockout Rlip genotypes, Figures S5–S17: Western blots showing target expression in Erbb2:Rlip−/−, Erbb2:Rlip+/−, and Erbb2:Rlip+/+ mice and in PyVT:Rlip−/−, PyVT:Rlip+/−, and PyVT:Rlip+/+ mice, Table S1: Statistical analyses comparing survival of Erbb2:Rlip−/−, Erbb2:Rlip+/−, and Erbb2:Rlip+/+ mice, Table S2: Statistical analyses comparing survival of PyVT:Rlip−/−, PyVT:Rlip+/−, and PyVT:Rlip+/+ mice, Supplemental Data S1: PyVT and LT_SV40 share sequence similarity.
