# Supplementary material for: Haploinsufficiency Interactions between RALBP1 and p53 in ERBB2 and PyVT Models of Mouse Mammary Carcinogenesis
Source: Cancers (Basel). 2021 Jul 2;13(13):3329. doi: 10.3390/cancers13133329 (PMC8268413; doi:10.3390/cancers13133329)
Supplement: Supplementary file 1 [file cancers-13-03329-s001.zip › cancers-1271595-supplementary.pdf]

Supplementary Materials

Haploinsufficiency Interactions between RALBP1 and p53 in ERBB2 and PyVT Models of Mouse Mammary Carcinogenesis

Sharda P. Singh , Jihyun Lee, Chhanda Bose, Hongzhi Li, Yate-Ching Yuan, Ashly Hindle, Sharad S. Singhal, Jonathan Kopel, Philip T. Palade, Catherine Jones, Rakhshanda L. Rahman and Sanjay Awasthi

Primer	Sequence (5'-3')	Tm (°C)	Product size (bp)
EGE-LBX-020-WT-F	AGTCAGTGCTCTGACCCCCTGAGC	65	WT:602bp
EGE-LBX-020-WT-R	TGGTAGAATCCATGTCTGCGGATGGG	63	
EGE-LBX-020-WT-F	AGTCAGTGCTCTGACCCCCTGAGC	65	WT:2603bp Mut:~633bp
EGE-LBX-020-3'MSD-R1	TTAGAGTGGGCACACCATCAGTCCC	63	

PCR Condition

Step	Temp.	Time	Cycles
1	95 ℃	3 min	1
2	95 ℃	30 sec	
3	62 ℃	30 sec	35
4	72 ℃	45 sec	
5	72 ℃	10 min	1
6	4 ℃	forever	1

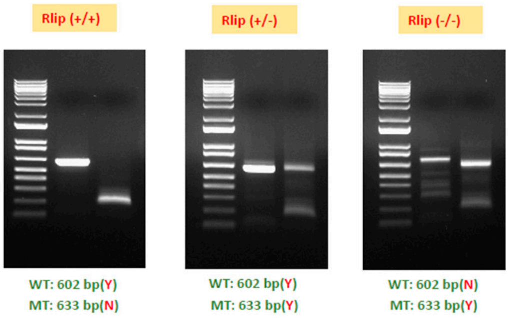

Figure S1. Genotyping details for *Ralpb1* wild-type and knockout genes as optimized by Biocytogen. Panels show the primer sequences, PCR thermal cycling protocol, and representative agarose gel images for each genotype.

Primer	Sequence (5'-3')
Transgene Forward	GGA AGC AAG TAC TTC ACA AGG G
Transgene Reverse	GGA AAG TCA CTA GGA GCA GGG
Internal Positive Control Forward	CAA ATG TTG CTT GTC TGG TG
Internal Positive Control Reverse	GTC AGT CGA GTG CAC AGT TT

EXPECTED RESULTS
Transgene = 556 bp
Internal positive control = 200 bp

PCR Condition

Step	Temp.	Time	Cycles
1	94 ℃	2 min	1
2	94 ℃	20 sec	10 (Touch down)
3	65 ℃	15sc -0.5C/cycle decrease	
4	68 ℃	10 sec	
5	94 ℃	15 sec	28
6	60 ℃	15 sec	
7	72 ℃	10 sec	
8	72 ℃	2 min	1
9	10 ℃	Hold	

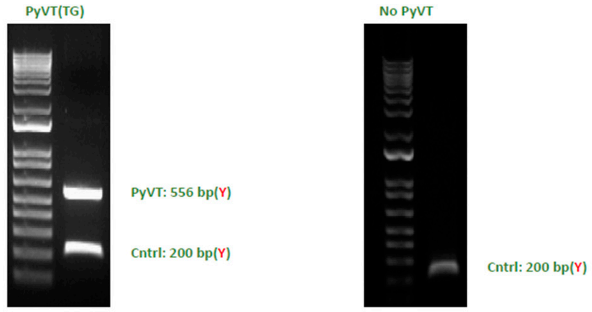

Figure S2. Genotyping details for the PyVT transgene as optimized by Jackson Laboratories. Panels show the primer sequences, PCR thermal cycling protocol, and representative agarose gel images for each genotype.

Primer	Sequence (5'-3')
Transgene Forward	CCC CGG GAG TAT GTG AGT GA
Transgene Reverse	TGA GCT GTT TTG AGG CTG ACA
Internal Positive Control Forward	CTA GGC CAC AGA ATT GAA AGA TCT
Internal Positive Control Reverse	GTA GGT GGA AAT TCT AGC ATC ATC C

EXPECTED RESULTS
Transgene = 79 bp
Internal positive control = 200 bp

PCR Condition

Step	Temp.	Time	Cycles
1	94 ℃	2 min	1
2	94 ℃	20 sec	10 (Touch down)
3	65 ℃	15sc -0.5C/cycle decrease	
4	68 ℃	10 sec	
5	94 ℃	15 sec	28
6	60 ℃	15 sec	
7	72 ℃	10 sec	
8	72 ℃	2 min	1
9	10 ℃	Hold	

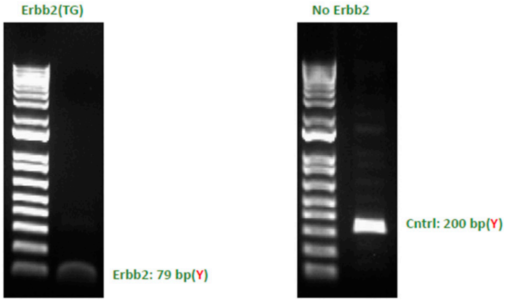

Figure S3. Genotyping details for the Erbb2 transgene as optimized by Jackson Laboratories. Panels show the primer sequences, PCR thermal cycling protocol, and representative agarose gel images for each genotype.

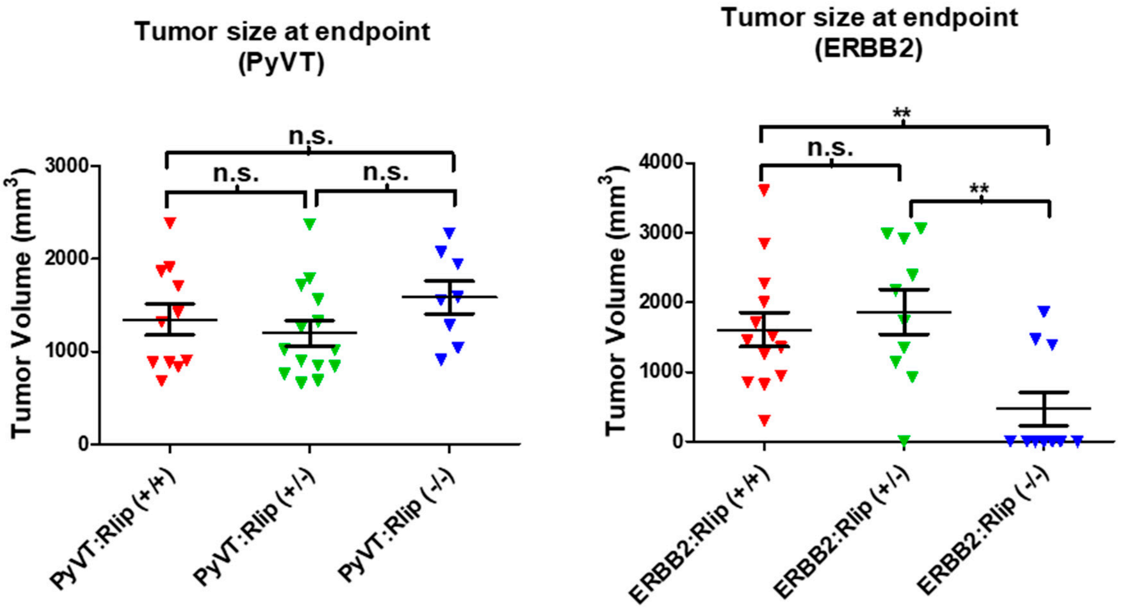

Figure S4. Tumor sizes at endpoint for PyVT and ERBB2 mice with wild-type, heterozygous knockout, or homozygous knockout Rlip genotypes. Note that 7 of 10 ERBB2:Rlip(−/−) mice were tumor-free at endpoint and were euthanized due to morbidities unrelated to tumor growth. The two asterisks (**) indicate $p < 0.01$, and n.s. indicates not significant.

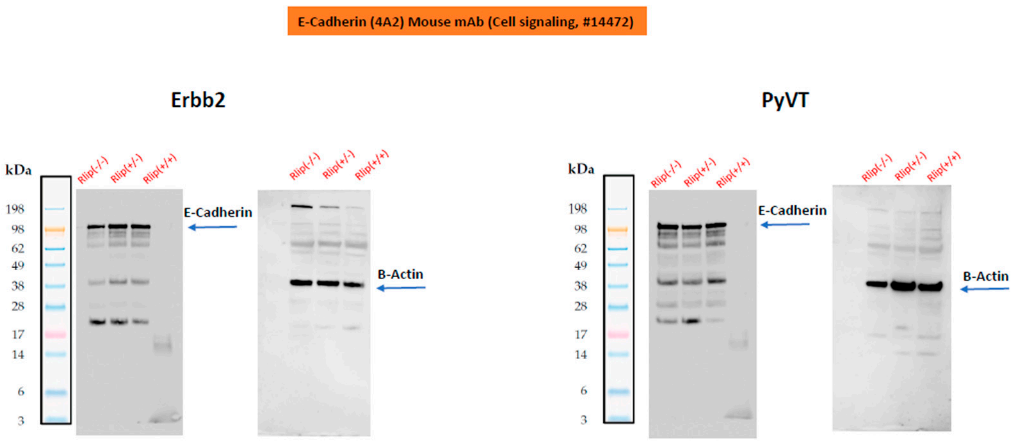

Figure S5. Western blots showing E-Cadherin expression in Erbb2:Rlip$^{-/-}$, Erbb2:Rlip$^{+/-}$, and Erbb2:Rlip$^{+/+}$ mice (left panels) and in PyVT:Rlip$^{-/-}$, PyVT:Rlip$^{+/-}$, and PyVT:Rlip$^{+/+}$ mice (right panels).

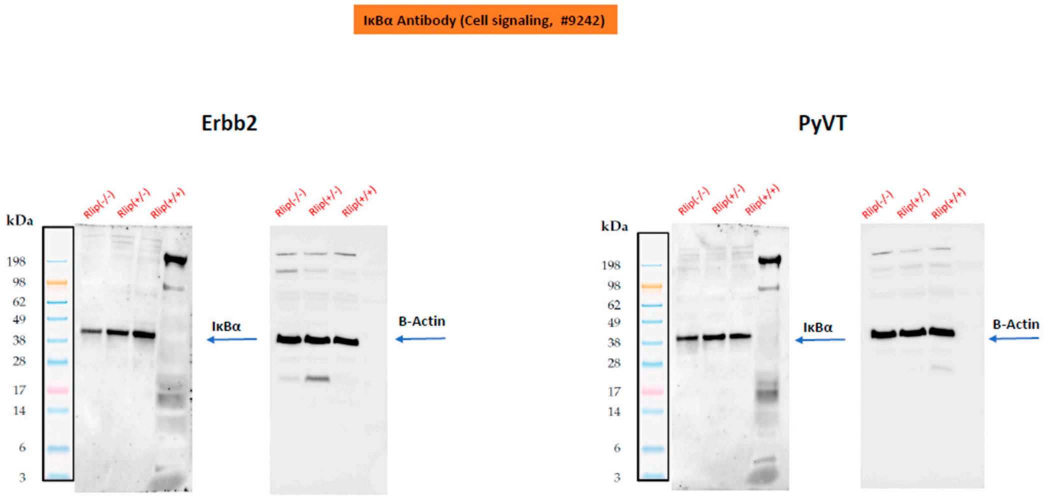

Figure S6. Western blots showing IκBα expression in Erbb2:Rlip$^{-/-}$, Erbb2:Rlip$^{+/-}$, and Erbb2:Rlip$^{+/+}$ mice (left panels) and in PyVT:Rlip$^{-/-}$, PyVT:Rlip$^{+/-}$, and PyVT:Rlip$^{+/+}$ mice (right panels).

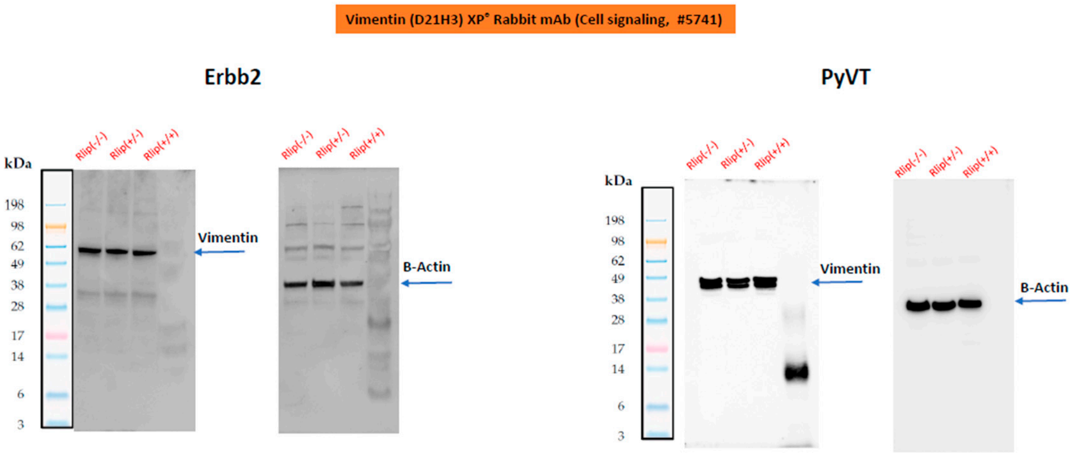

Figure S7. Western blots showing Vimentin expression in Erbb2:Rlip$^{-/-}$, Erbb2:Rlip$^{+/-}$, and Erbb2:Rlip$^{+/+}$ mice (left panels) and in PyVT:Rlip$^{-/-}$, PyVT:Rlip$^{+/-}$, and PyVT:Rlip$^{+/+}$ mice (right panels).

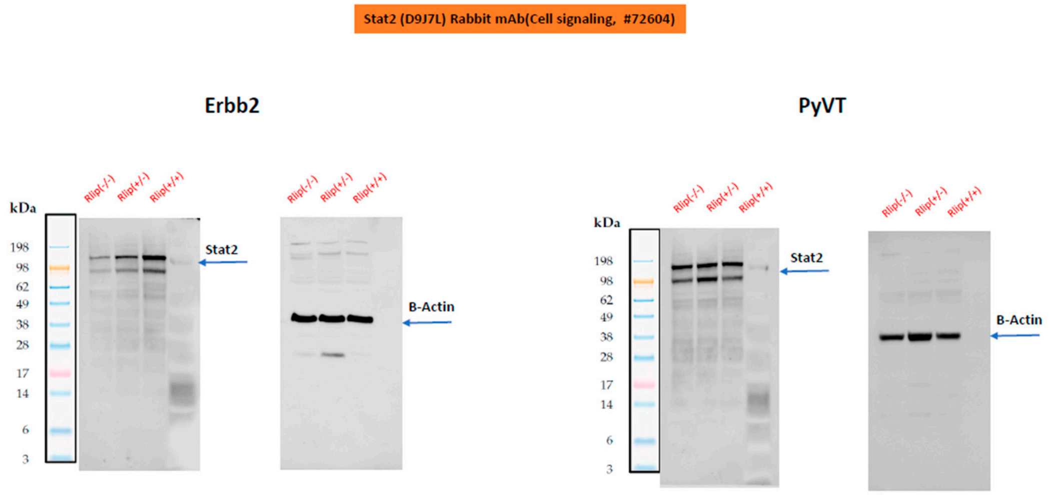

Figure S8. Western blots showing Stat2 expression in Erbb2:Rlip$^{-/-}$, Erbb2:Rlip$^{+/-}$, and Erbb2:Rlip$^{+/+}$ mice (left panels) and in PyVT:Rlip$^{-/-}$, PyVT:Rlip$^{+/-}$, and PyVT:Rlip$^{+/+}$ mice (right panels).

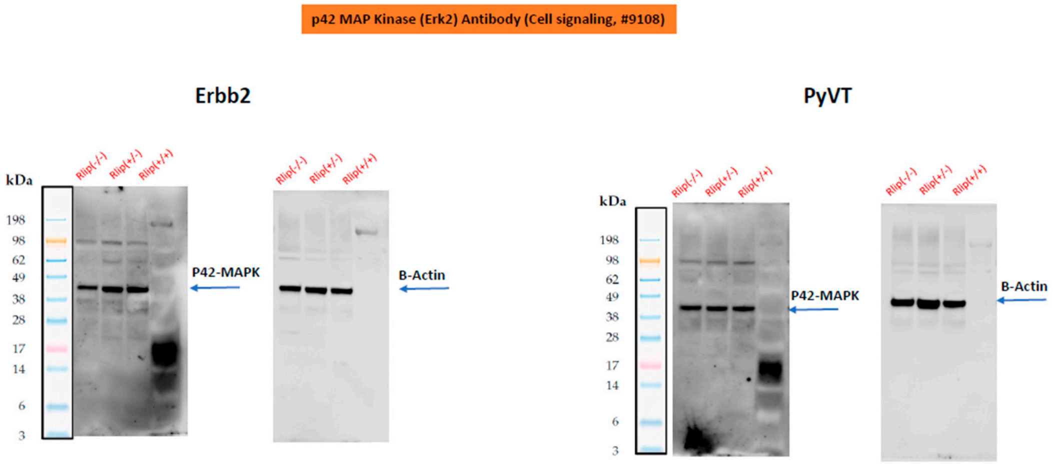

Figure S9. Western blots showing p42 MAP Kinase (Erk2) expression in Erbb2:Rlip$^{-/-}$, Erbb2:Rlip$^{+/-}$, and Erbb2:Rlip$^{+/+}$ mice (left panels) and in PyVT:Rlip$^{-/-}$, PyVT:Rlip$^{+/-}$, and PyVT:Rlip$^{+/+}$ mice (right panels).

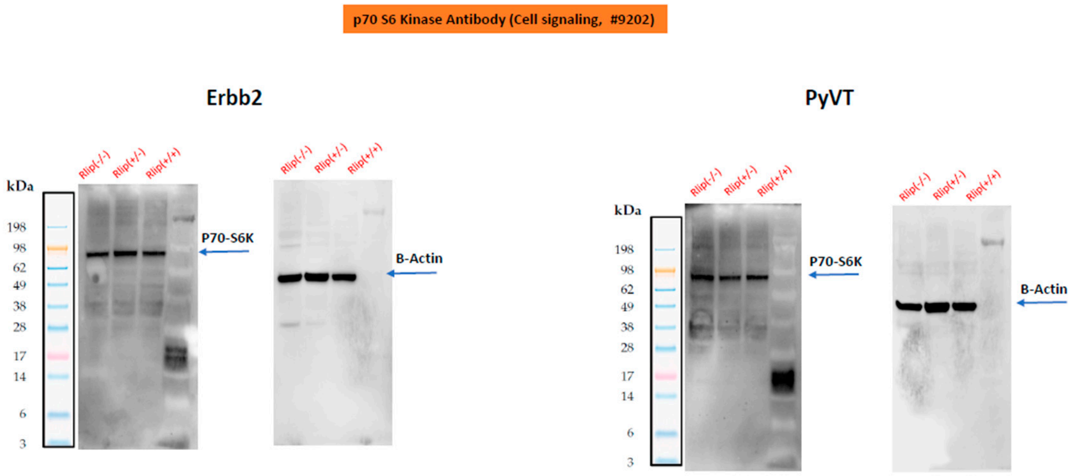

Figure S10. Western blots showing p70 S6 Kinase expression in Erbb2:Rlip$^{-/-}$, Erbb2:Rlip$^{+/-}$, and Erbb2:Rlip$^{+/+}$ mice (left panels) and in PyVT:Rlip$^{-/-}$, PyVT:Rlip$^{+/-}$, and PyVT:Rlip$^{+/+}$ mice (right panels).

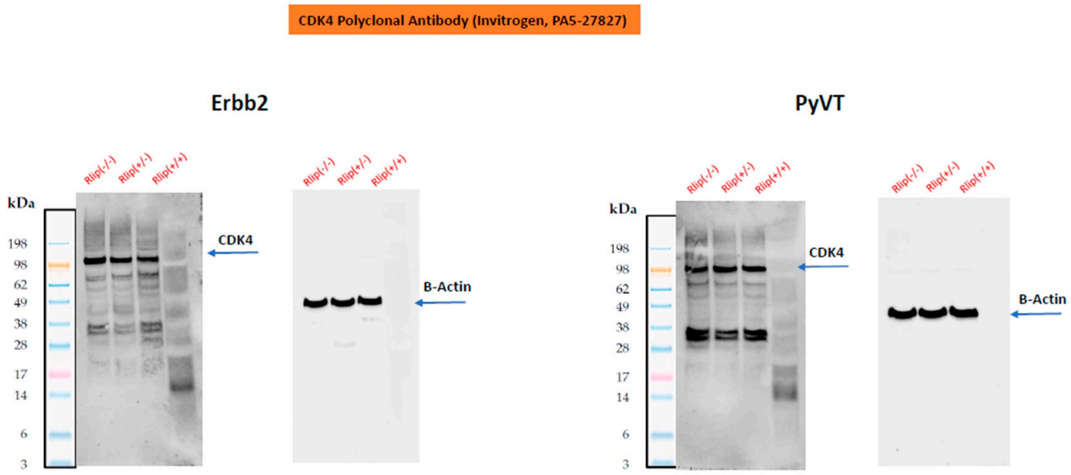

Figure S11. Western blots showing CDK4 expression in Erbb2:Rlip$^{-/-}$, Erbb2:Rlip$^{+/-}$, and Erbb2:Rlip$^{+/+}$ mice (left panels) and in PyVT:Rlip$^{-/-}$, PyVT:Rlip$^{+/-}$, and PyVT:Rlip$^{+/+}$ mice (right panels).

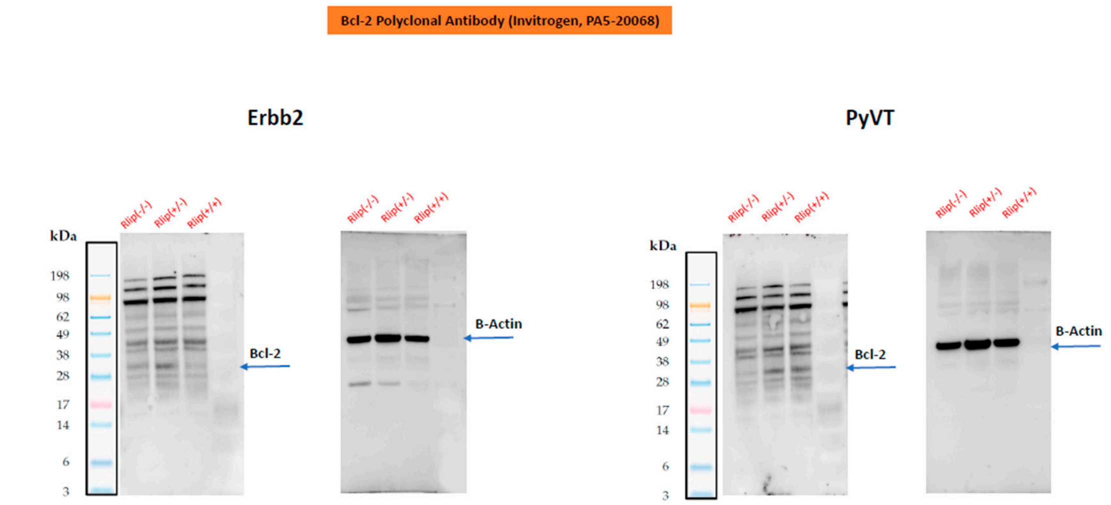

Figure S12. Western blots showing Bcl-2 expression in Erbb2:Rlip$^{-/-}$, Erbb2:Rlip$^{+/-}$, and Erbb2:Rlip$^{+/+}$ mice (left panels) and in PyVT:Rlip$^{-/-}$, PyVT:Rlip$^{+/-}$, and PyVT:Rlip$^{+/+}$ mice (right panels).

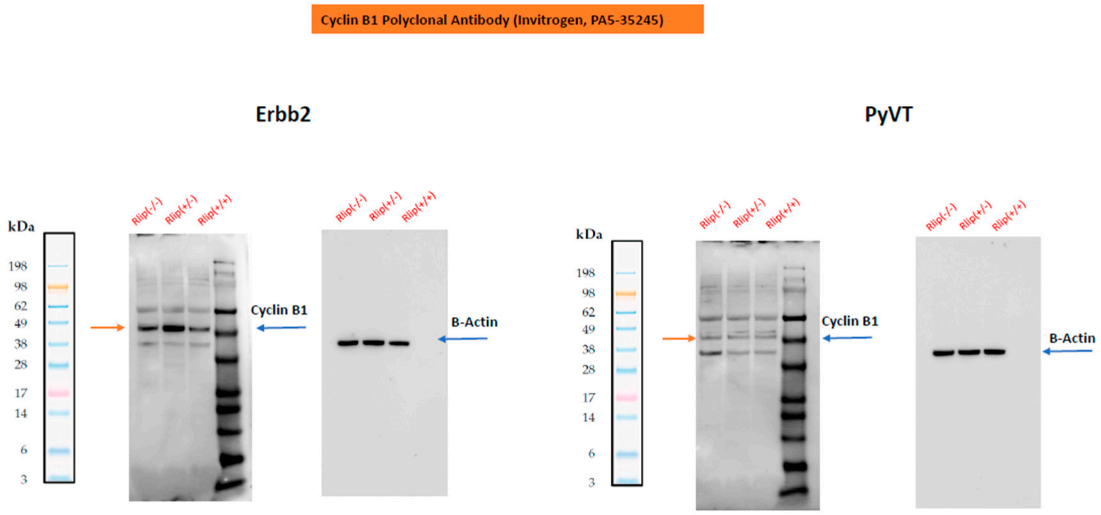

Figure S13. Western blots showing Cyclin B1 expression in Erbb2:Rlip$^{-/-}$, Erbb2:Rlip$^{+/-}$, and Erbb2:Rlip$^{+/+}$ mice (left panels) and in PyVT:Rlip$^{-/-}$, PyVT:Rlip$^{+/-}$, and PyVT:Rlip$^{+/+}$ mice (right panels).

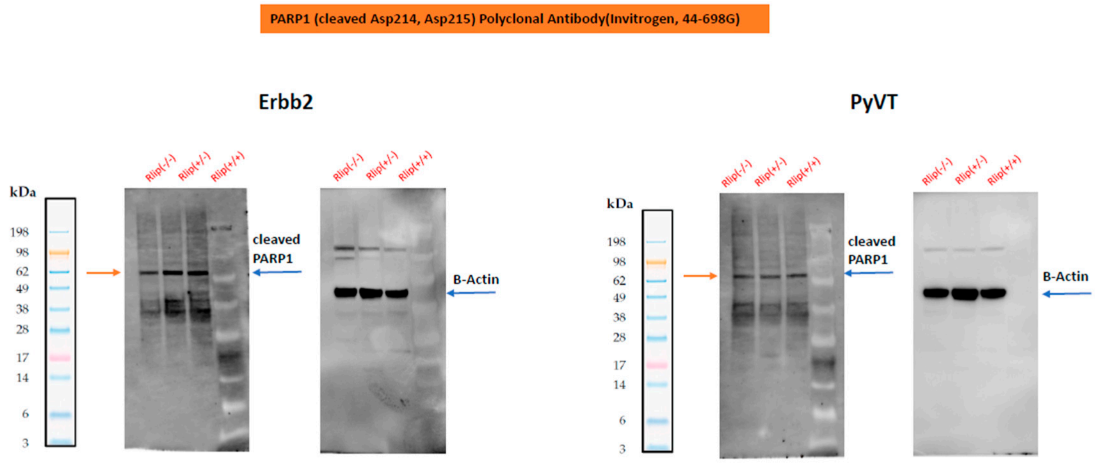

Figure S14. Western blots showing cleaved PARP1 expression in Erbb2:Rlip$^{-/-}$, Erbb2:Rlip$^{+/-}$, and Erbb2:Rlip$^{+/+}$ mice (left panels) and in PyVT:Rlip$^{-/-}$, PyVT:Rlip$^{+/-}$, and PyVT:Rlip$^{+/+}$ mice (right panels). The indicated band is the 62 kDa fragment.

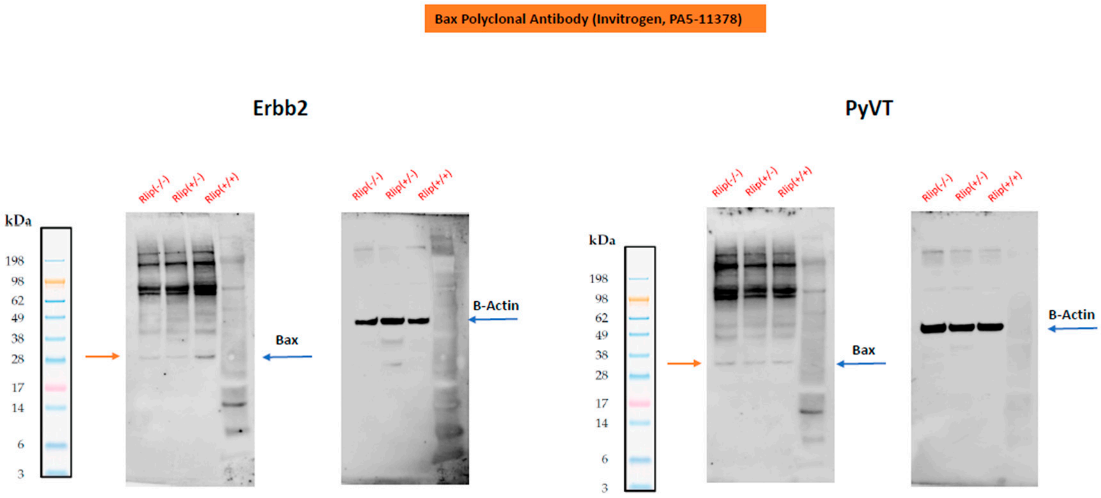

Figure S15. Western blots showing Bax expression in Erbb2:Rlip$^{-/-}$, Erbb2:Rlip$^{+/-}$, and Erbb2:Rlip$^{+/+}$ mice (left panels) and in PyVT:Rlip$^{-/-}$, PyVT:Rlip$^{+/-}$, and PyVT:Rlip$^{+/+}$ mice (right panels).

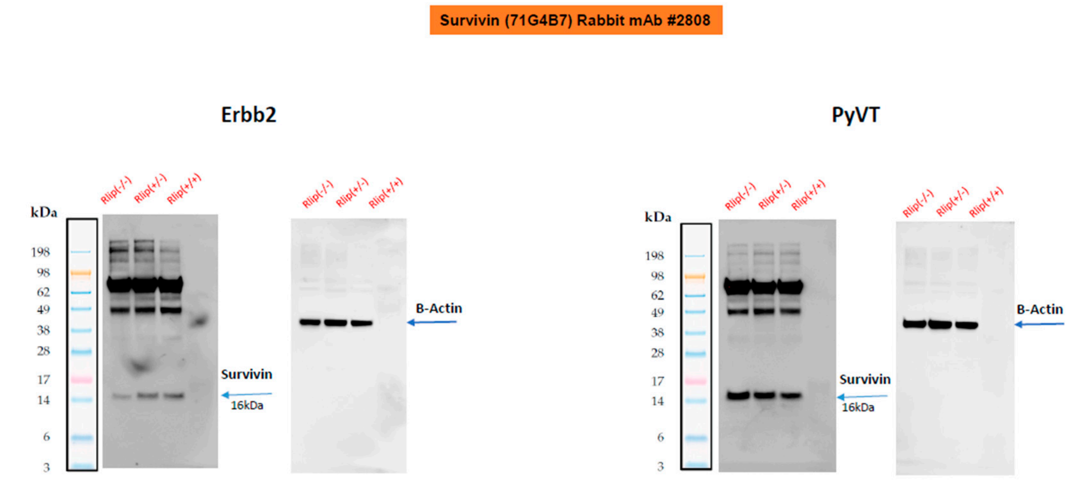

Figure S16. Western blots showing Survivin expression in Erbb2:Rlip$^{-/-}$, Erbb2:Rlip$^{+/-}$, and Erbb2:Rlip$^{+/+}$ mice (left panels) and in PyVT:Rlip$^{-/-}$, PyVT:Rlip$^{+/-}$, and PyVT:Rlip$^{+/+}$ mice (right panels).

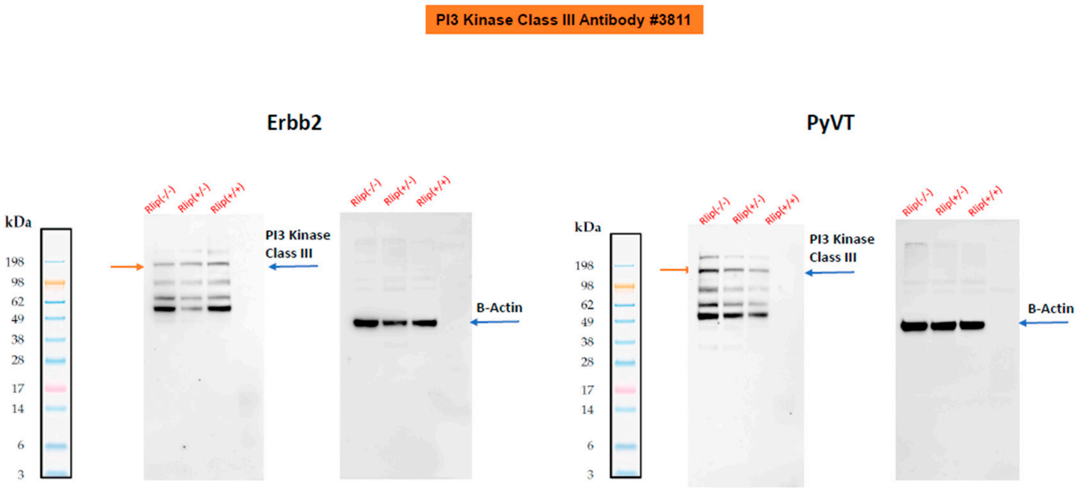

Figure S17. Western blots showing PI3 Kinase Class III expression in Erbb2:Rlip$^{-/-}$, Erbb2:Rlip$^{+/-}$, and Erbb2:Rlip$^{+/+}$ mice (left panels) and in PyVT:Rlip$^{-/-}$, PyVT:Rlip$^{+/-}$, and PyVT:Rlip$^{+/+}$ mice (right panels).

Table S1. Statistical analyses comparing survival of Erbb2:Rlip$^{-/-}$, Erbb2:Rlip$^{+/-}$, and Erbb2:Rlip$^{+/+}$ mice. Output is from GraphPad Prism 9.0 for Windows. Median survival is in days. ** indicates p < 0.01; *** indicates p < 0.001.

Comparison of Survival Curves Erbb2 Mice			
Log-rank (Mantel-Cox) test (recommended)			
Chi square			17.04
df			2
p value			0.0002
p value summary			***
Are the survival curves sig different?			Yes
Logrank test for trend (recommended)			
Chi square			6.664
df			1
p value			0.0098
p value summary			**
Sig. trend?			Yes
Gehan-Breslow-Wilcoxon test			
Chi square			13.43
df			2
p value			0.0012
p value summary			**
Are the survival curves sig different?			Yes
	**Rlip-WT/Erbb2**	**Rlip-Het/Erbb2**	**Rlip-Null/Erbb2**
Number of rows	36	36	36
# of blank lines	23	26	25
# rows with impossible data	0	0	0
# censored subjects	0	0	0
# deaths/events	13	10	11
Median survival (Days)	236	461.5	326

Table S2. Statistical analyses comparing survival of PyVT:Rlip$^{-/-}$, PyVT:Rlip$^{+/-}$, and PyVT:Rlip$^{+/+}$ mice. Output is from GraphPad Prism 9.0 for Windows. Median survival is in days. ns (p>0.1) indicates not significant.

Comparison of Survival Curves of PyVT Mice			
Log-rank (Mantel-Cox) test (recommended)			
Chi square			3.790
df			2
p value			0.1503
p value summary			ns
Are the survival curves sig different?			No
Logrank test for trend (recommended)			
Chi square			0.8180
df			1
p value			0.3658
p value summary			ns
Sig. trend?			No
Gehan-Breslow-Wilcoxon test			
Chi square			2.818
df			2
p value			0.2444
p value summary			ns
Are the survival curves sig different?			No
	**Rlip-WT/PyVT**	**Rlip-Het/PyVT**	**Rlip-Null/PyVT**

Number of rows	40	40	40
# of blank lines	26	28	29
# rows with impossible data	0	0	1
# censored subjects	0	0	0
# deaths/events	14	12	10
Median survival (Days)	133	127	133.5

```
LT_SV40            1 MDKVLNREESLQLMDLLGLERSAWGNIPLMRKAYLKKCKEFHPDKGGDEE     50
                     ||:||:|.:..:|::||.|.|..||:...|::||.::....||||||...
MT_POVBG           1 MDRVLSRADKERLLELLKLPRQLWGDFGRMQQAYKQQSLLLHPDKGGSHA     50

LT_SV40           51 KMKKMNTLYKKMEDGVKYAHQPDFGGF-----------WDATEIPTYGTD     89
                     .|:::|:|:...:..| |..:.:.||.           |:.:...|:|
MT_POVBG          51 LMQELNSLWGTFKTEV-YNLRMNLGGTGFQVRRLHADGWNLSTKDTFG--     97

LT_SV40           90 EWEQWWNAFNEENLFCSEEMPSSDDE---ATADSQHSTPPKKKRKVEDPK    136
                       ::::..|....|.|...:..|...   .....||       |:::|..
MT_POVBG          98 --DRYYQRFCRMPLTCLVNVKYSSCSCILCLLRKQH-------RELKDKC    138

LT_SV40          137 DFPSELLSFLSHAVFSNRTLACFAI------YTTKEKAAL-LYKKIMEKY    179
                     |....:|.            .||.:      :.|..:..| ||...:...
MT_POVBG         139 DARCLVLG------------ECFCLECYMQWFGTPTRDVLNLYADFIASM    176

LT_SV40          180 SVTFISR--HNSYNHNILFFLTPHRHRVSAINNYAQKLCTFSFLICKG--    225
                     .:.::..  |:.||        |.|.     :...::..|..:.:..|
MT_POVBG         177 PIDWLDLDVHSVYN--------PKRR-----SEELRRAATVHYTMTTGHS    213

LT_SV40          226 ---------------------VNKEYLMYSALTRDPFSVIEESLPGGLKE    254
                                          .::...:.|.|:...:||        ::.
MT_POVBG         214 AMEASTSQGNGMISSESGTPATSRRLRLPSLLSNPTYSV--------MRS    255

LT_SV40          255 HDFNPEEAEETKQVSWKLVTEYAMETKCDDVLLLLG------------MY    292
                     |.:.|...  .:|:...::.|.      |::|:||.            :|
MT_POVBG         256 HSYPPTRV--LQQIHPHILLEE------DEILVLLSPMTAYPRTPPELLY    297

LT_SV40          293 LEFQYSFEMCLKCIKKEQPSHYKYHEKHYANAAIFADSKNQKTICQQAVD    342
                     .|   |.:..|:.:::|:       |::.....::.|...::.:.|....
MT_POVBG         298 PE---SDQDQLEPLEEEE-------EEYMPMEDLYLDILPEEQVPQLIPP    337

LT_SV40          343 TVLAKKRVDSLQ-LTREQMLTNRFNDLLDRMDIMFGSTGSA---------    382
                     .::.:..:...: |....:....|:.:||....|..:..:|
MT_POVBG         338 PIIPRAGLSPWEGLILRDLQRAHFDPILDASQRMRATHRAALRAHSMQRH    387

LT_SV40          383 --DIEEWMAGVAWLHCLLPKMDSVVYDFLKCMVYNIPKKRYWLFKGPIDS    430
                       .:...:..|.:|..||.          .|::..|..||...|
MT_POVBG         388 LRRLGRTLLLVTFLAALLG----------ICLMLFILIKRSRHF------    421

LT_SV40          431 GKTTLAAALLELCGGKALNVNLPLDRLNFELGVAIDQFLVVFEDVKGTGG    480

MT_POVBG         422 --------------------------------------------------    421
```

Supplemental Data S1. PyVT and LT_SV40 share sequence similarity. From EMBOSS Needle # Length: 778; # Identity: 89/778 (11.4%); # Similarity: 177/778 (22.8%); # Gaps: 427/778 (54.9%); #Score: 126.0.

© 2021 by the authors. Licensee MDPI, Basel, Switzerland. This article is an open access article distributed under the terms and conditions of the Creative Commons Attribution (CC BY) license (http://creativecommons.org/licenses/by/4.0/).
